# Performance of five automated white matter hyperintensity segmentation methods in a multicenter dataset

**DOI:** 10.1038/s41598-019-52966-0

**Published:** 2019-11-14

**Authors:** Rutger Heinen, Martijn D. Steenwijk, Frederik Barkhof, J. Matthijs Biesbroek, Wiesje M. van der Flier, Hugo J. Kuijf, Niels D. Prins, Hugo Vrenken, Geert Jan Biessels, Jeroen de Bresser, TRACE-VCI study group, E. van den Berg, G. J. Biessels, J. M. F. Boomsma, L. G. Exalto, D. A. Ferro, C. J. M. Frijns, O. N. Groeneveld, R. Heinen, N. M. van Kalsbeek, J. H. Verwer, J. de Bresser, H. J. Kuijf, M. E. Emmelot-Vonk, H. L. Koek, M. R. Benedictus, J. Bremer, W. M. van der Flier, A. E. Leeuwis, J. Leijenaar, N. D. Prins, P. Scheltens, B. M. Tijms, F. Barkhof, M. P. Wattjes, C. E. Teunissen, T. Koene, J. M. F. Boomsma, H. C. Weinstein, M. Hamaker, R. Faaij, M. Pleizier, M. Prins, E. Vriens

**Affiliations:** 1 Department of Neurology and Neurosurgery, UMC Utrecht Brain Center, University Medical Center Utrecht, Utrecht University, Utrecht, The Netherlands; 2 Department of Anatomy and Neurosciences, Amsterdam Neuroscience, Vrije Universiteit Amsterdam, Amsterdam UMC, Amsterdam, The Netherlands; 3 Department of Radiology and Nuclear Medicine, Amsterdam Neuroscience, Vrije Universiteit Amsterdam, Amsterdam UMC, Amsterdam, The Netherlands; 4 Institutes of Neurology & Healthcare Engineering, University College London (UCL), London, United Kingdom; 5 Alzheimer Center & Department of Neurology, Vrije Universiteit Amsterdam, Amsterdam UMC, Amsterdam, The Netherlands; 6 Department of Epidemiology and Biostatistics, Vrije Universiteit Amsterdam, Amsterdam UMC, Amsterdam, The Netherlands; 7 Image Sciences Institute, University Medical Center Utrecht, Utrecht, The Netherlands; 8 Department of Radiology, Leiden University Medical Center, Leiden, The Netherlands; 9 Department of Neurology, University Medical Center Utrecht, Utrecht University, Utrecht, The Netherlands; 10 Department of Radiology, University Medical Center Utrecht, Utrecht University, Utrecht, The Netherlands; 11 Image Sciences Institute, University Medical Center Utrecht, Utrecht University, Utrecht, The Netherlands; 12 Department of Geriatrics, University Medical Center Utrecht, Utrecht University, Utrecht, The Netherlands; 13 Alzheimer Center and Department of Neurology, Amsterdam UMC, Vrije Universiteit Amsterdam, Amsterdam, The Netherlands; 14 Department of Radiology and Nuclear Medicine, Amsterdam UMC, Vrije Universiteit Amsterdam, Amsterdam, The Netherlands; 15 Department of Clinical Chemistry, Amsterdam UMC, Vrije Universiteit Amsterdam, Amsterdam, The Netherlands; 16 Department of Medical Psychology, Amsterdam UMC, Vrije Universiteit Amsterdam, Amsterdam, The Netherlands; 17 Department of Neurology, Onze Lieve Vrouwe Gasthuis West, Amsterdam, The Netherlands; 18 Hospital Diakonessenhuis, Zeist, The Netherlands

**Keywords:** Stroke, Stroke

## Abstract

White matter hyperintensities (WMHs) are a common manifestation of cerebral small vessel disease, that is increasingly studied with large, pooled multicenter datasets. This data pooling increases statistical power, but poses challenges for automated WMH segmentation. Although there is extensive literature on the evaluation of automated WMH segmentation methods, such evaluations in a multicenter setting are lacking. We performed WMH segmentations in sixty patients scanned on six different magnetic resonance imaging (MRI) scanners (10 patients per scanner) using five freely available and fully-automated WMH segmentation methods (Cascade, kNN-TTP, Lesion-TOADS, LST-LGA and LST-LPA). Different MRI scanner vendors and field strengths were included. We compared these automated WMH segmentations with manual WMH segmentations as a reference. Performance of each method both within and across scanners was assessed using spatial and volumetric correspondence with the reference segmentations by Dice’s similarity coefficient (DSC) and intra-class correlation coefficient (ICC) respectively. We found the best performance, both within and across scanners, for kNN-TTP, followed by LST-LPA and LST-LGA, with worse performance for Lesion-TOADS and Cascade. Our findings can serve as a guide for choosing a method and also highlight the importance to further improve and evaluate consistency of methods in a multicenter setting.

## Introduction

Pooling of multicenter brain magnetic resonance imaging (MRI) data is a trend in various research fields, including studies on ageing related brain diseases^1–3^. Pooling of multicenter data increases sample size (and thus statistical power) and can support a faster patient inclusion. Moreover, findings of multicenter studies may have a larger external validity and are more readily translatable to a clinical setting. However, pooling of brain MRI data poses challenges in automated segmentation due to variations in image acquisition.

White matter hyperintensities of presumed vascular origin (WMHs) are frequently encountered in studies on ageing related brain diseases. Achieving accurate and precise WMH segmentations can be challenging across MRI scanners of different vendors, field strengths and scan protocols. Variability in MRI acquisition can lead to differences in the contrast and borders of WMHs and thereby quantification bias^4–6^.

Several automated and semi-automated methods to segment WMHs currently exist, using various algorithms that rely on intensity, spatial information, or both^5^. These methods can be broadly classified as supervised (i.e. trained using manual segmentations as a refs^7,8^), unsupervised (without training^9–11^) and semi-supervised (with only a small portion of the available data used for training^12^. A recent study provided an extensive overview of existing supervised, unsupervised and semi-supervised methods^13^. Challenges for these methods include false positive (e.g. artefacts, infarcts) and false negative (often for punctate lesions) results. Other challenges include dealing with varying WMH lesion loads (usually lower in MS than in patients with WMHs of presumed vascular origin) and with co-occurring pathologies (e.g. extensive atrophy). There is extensive literature on the evaluation of WMH segmentation methods in different settings, also addressing these challenges^4^. However, the performance of such methods is typically evaluated on single center, single scanner datasets. For WMHs of presumed vascular origin, there is a lack of studies comparing performance of these methods in multicenter, multiscanner datasets and this is an important knowledge gap^4,14^.

Therefore, the present study aimed to assess performance, in terms of spatial and volumetric correspondence with reference segmentations, of five automated WMH segmentation methods in a multicenter, multiscanner dataset of patients with WMHs of presumed vascular origin. In particular, we also addressed which methods showed variation in performance across scanners. In addition, we assessed if performance was dependent on WMH lesion load. To this end, we selected five methods that were fully automatic and freely available for academic research: Cascade^15,16^, k-nearest neighbor classification with tissue type priors (kNN-TTP)^17^, Lesion-TOpology-preserving Anatomical Segmentation (Lesion-TOADS)^11^, the Lesion Segmentation Tool Lesion Prediction Algorithm (LST-LPA) and the Lesion Segmentation Tool Lesion Growth Algorithm (LST-LGA)^10^.

## Results

### Reference segmentations

The reference segmentations showed a very good inter-rater agreement regarding spatial (Dice’s similarity coefficient (DSC) ± standard deviation (SD): 0.80 ± 0.09) and volumetric agreement (Intra-class correlation coefficient (ICC): 0.97). The intra-rater agreement (DSC ± SD: 0.80 ± 0.08; ICC: 0.99) was also very good. In the test set, seventeen subjects had a Fazekas rating of 1, eighteen subjects had a 2, and seven subjects had a 3. The mean WMH volume (±SD) was 21 ± 10 mL with a median of 10 mL and volumes per patient ranging from 0.9 to 199 mL (see Table 1).

**Table 1:** Mean WMH volume of the reference segmentations and the segmentations of the methods for each scanner (n = 42; n = 7 per scanner).

WMH volume	GE Signa HDxt 1.5T	GE Signa HDxt 3T	GE Discovery MR750 3T	Philips Ingenuity 3T	Philips Ingenia 3T	Philips Achieva 3T	Overall mean ± SD
Reference	22 ± 31	16 ± 18	9 ± 10	14 ± 17	41 ± 71	24 ± 26	**21 **±** 10**
Cascade	26 ± 20	19 ± 11	13 ± 5	19 ± 10	12 ± 4	11 ± 5	**17 **±** 5**
kNN-TTP	16 ± 19	14 ± 13	9 ± 10	14 ± 17	32 ± 49	20 ± 22	**18 **±** 7**
Lesion-TOADS	19 ± 20	16 ± 12	11 ± 9	36 ± 24	30 ± 45	31 ± 16	**24 **±** 9**
LST-LGA	20 ± 19	19 ± 23	12 ± 15	15 ± 20	22 ± 28	14 ± 17	**17 **±** 4**
LST-LPA	18 ± 22	15 ± 18	11 ± 13	14 ± 18	33 ± 51	18 ± 22	**18 **±** 7**

Note: Values represent mean WMH volumes ± SD in mL. Reference: reference segmentations.

### Quality assessment

**Figure 1:**
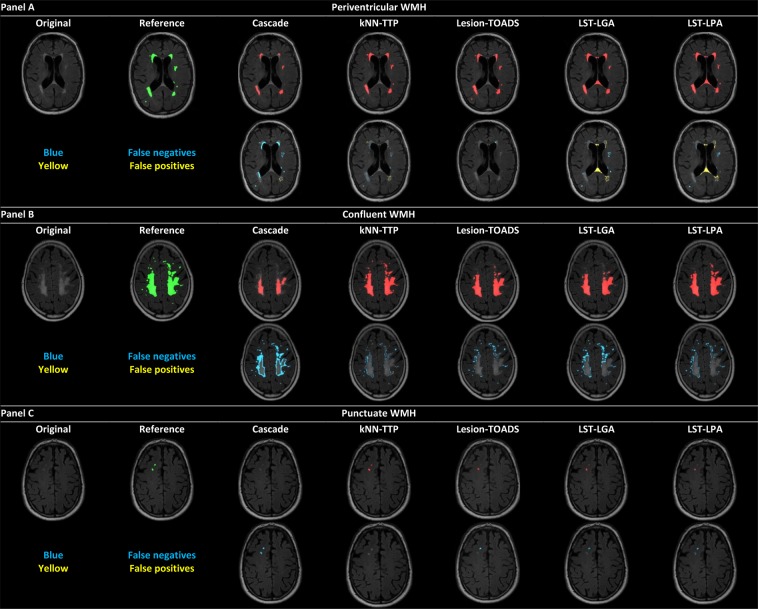
WMH segmentations of the methods regarding periventricular, confluent and punctuate WMHs. Example of WMH segmentations for a subject (subject A) with predominantly periventricular WMHs (panel A), a subject (subject B) with large confluent WMHs (panel B) and a subject (subject C) with predominantly punctuate WMHs (panel C). Top rows panels (A–C) original FLAIR scan and WMH reference segmentation (green) and WMH segmentations of all methods (red) are shown. Bottom rows panels (A–C) false negative voxels are shown in blue; false positive voxels are shown in yellow.

Examples of the automated WMH segmentation results are shown in Fig. 1. Several differences between methods can be visually appreciated. For example, methods seemed to differ on how they segment (over or under) different types of WMHs (i.e. periventricular, confluent and punctuate WMHs). Also, the nature of segmentation errors varied between methods (i.e. false-positive (FP) versus false-negative (FN) WMH voxels: see Fig. 1). In a quantitative analysis, kNN-TTP showed the lowest mean FP and FN volumes (mean FP volume ± SD/mean FN volume ± SD: 2 ± 2/5 ± 11 mL), followed by LST-LPA (4 ± 4/6 ± 10 mL), LST-LGA (5 ± 5/8 ± 19 mL). Cascade showed a lower mean FP volume (8 ± 7 mL) but higher mean FN volume (12 ± 29 mL) than Lesion-TOADS (10 ± 16/7 ± 12 mL).

### Performance of WMH segmentation methods

Performance of each method, both within and averaged across all scanners, is shown in Table 2. The highest mean performance across scanners was seen for kNN-TTP, both in terms of spatial correspondence with the reference segmentations (mean DSC ± SD: 0.73 ± 0.03) as in terms of volumetric correspondence with the reference segmentations (mean ICC ± SD: 0.97 ± 0.02) (see Table 2). LST-LPA showed a slightly lower performance in terms of volumetric correspondence (mean ICC ± SD: 0.92 ± 0.03) and performed less than kNN-TTP in terms of spatial correspondence (mean DSC ± SD: 0.60 ± 0.06). The mean absolute WMH volume differences between the methods and the reference segmentations were also lowest for kNN-TTP (5 ± 3 mL; percentage of the mean WMH volume of the reference segmentations: 24%) and LST-LPA (5 ± 2 mL; 24%) (see Table 2). Both methods did show a tendency for slight underestimation of the WMH volume compared to the reference segmentations. LST-LGA showed a performance comparable to LST-LPA (mean DSC ± SD: 0.57 ± 0.03; mean ICC ± SD: 0.65 ± 0.29) but with a larger mean absolute WMH volume difference (8 ± 5 mL; 38%). Performance was lower for Lesion-TOADS (0.53 ± 0.08/0.65 ± 0.29) and Cascade (0.40 ± 0.05/0.44 ± 0.01) with also markedly higher mean absolute WMH volume differences for both methods (Lesion-TOADS: 12 ± 8 mL; 57%; Cascade: 16 ± 7 mL; 76%) (see Table 2).

**Table 2:** Performance of the WMH segmentation methods compared to the reference segmentations (n = 42; n = 7 per scanner).

Method	Measure	GE Signa HDxt 1.5T	GE Signa HDxt 3T	GE Discovery MR750 3T	Philips Ingenuity 3T	Philips Ingenia 3T	Philips Achieva 3T	Overall mean ± SD
Ref	WMH	22 ± 31	16 ± 18	9 ± 10	14 ± 17	41 ± 71	24 ± 26	**21 ± 10**
Cascade	ΔWMH	4 ± 15	4 ± 19	4 ± 11	6 ± 12	−29 ± 68	−13 ± 22	**−4 ± 13**
	|ΔWMH|	12 ± 9	14 ± 12	10 ± 5	11 ± 6	32 ± 66	15 ± 21	**16 ± 7**
	DSC	0.48 ± 0.29	0.35 ± 0.20	0.34 ± 0.25	0.43 ± 0.22	0.40 ± 0.21	0.41 ± 0.14	**0**.**40 ± 0**.**05**
	ICC	0.45 (−0.19; 0.87)	0.45 (−0.18; 0.87)	*	0.44 (−0.16; 0.86)	0.43 (−0.40; 0.87)	0.46 (−0.32; 0.88)	**0**.**44 ± 0**.**01**
kNN-TTP	ΔWMH	−5 ± 13	−2 ± 7	0.8 ± 3	0.9 ± 2	−9 ± 22	−4 ± 4	−**3 ± 4**
	|ΔWMH|	6 ± 13	5 ± 6	2 ± 2	1 ± 2	10 ± 21	4 ± 4	**5 ± 3**
	DSC	0.74 ± 0.11	0.68 ± 0.11	0.71 ± 0.12	0.74 ± 0.10	0.75 ± 0.14	0.76 ± 0.07	**0**.**73 ± 0**.**03**
	ICC	0.99 (0.94; 1.00)	0.95 (0.73; 0.99)	0.97 (0.76; 0.99)	0.96 (0.80; 0.99)	0.99 (0.95; 1.00)	0.98 (0.88; 1.00)	**0**.**97 ± 0**.**02**
Lesion-TOADS	ΔWMH	−3 ± 10	0.5 ± 9	2 ± 3	23 ± 31	−11 ± 26	7 ± 24	**3 ± 10**
	|ΔWMH|	5 ± 9	6 ± 6	3 ± 2	25 ± 29	14 ± 24	16 ± 18	**12 ± 8**
	DSC	0.63 ± 0.21	0.56 ± 0.20	0.49 ± 0.22	0.43 ± 0.34	0.61 ± 0.15	0.46 ± 0.32	**0**.**53 ± 0**.**08**
	ICC	0.80 (0.28; 0.96)	0.77 (0.22; 0.96)	0.69 (−0.01; 0.94)	*	0.93 (0.65; 0.99)	0.08 (−0.54; 0.73)	**0**.**65 ± 0**.**29**
LST-LGA	ΔWMH	−2 ± 13	4 ± 7	4 ± 6	2 ± 4	−19 ± 44	−10 ± 10	**−4 ± 8**
	|ΔWMH|	7 ± 11	6 ± 6	4 ± 5	3 ± 2	19 ± 44	10 ± 10	**8 ± 5**
	DSC	0.58 ± 0.16	0.53 ± 0.18	0.54 ± 0.12	0.53 ± 0.17	0.63 ± 0.18	0.59 ± 0.11	**0**.**57 ± 0**.**03**
	ICC	0.95 (0.70; 0.99)	0.92 (0.62; 0.99)	0.97 (0.78; 1.00)	0.92 (0.61; 0.99)	0.90 (0.32; 0.98)	0.89 (−0.03; 0.99)	**0**.**92 ± 0**.**03**
LST-LPA	ΔWMH	−3 ± 10	−0.2 ± 7	2 ± 5	0.6 ± 4	−8 ± 21	-−6 ± 6	**−2 ± 4**
	|ΔWMH|	5 ± 8	4 ± 5	3 ± 5	3 ± 2	10 ± 20	7 ± 5	**5 ± 2**
	DSC	0.65 ± 0.13	0.52 ± 0.20	0.53 ± 0.17	0.59 ± 0.17	0.69 ± 0.15	0.63 ± 0.11	**0**.**60 ± 0**.**06**
	ICC	0.97 (0.85; 1.00)	0.87 (0.47; 0.98)	0.94 (0.71; 0.99)	0.88 (0.43; 0.98)	0.96 (0.80; 0.99)	0.93 (0.54; 0.99)	**0**.**92 ± 0**.**04**

Note: WMH, ΔWMH, |ΔWMH| and DSC are shown as means ± SD. ICC is shown with 95% confidence interval.

Ref: Reference; WMH: WMH volume (mL); ΔWMH: difference in WMH volume (mL) between the reference segmentations and segmentations of the methods; |ΔWMH|: absolute difference in WMH volume (mL) between the reference segmentations and segmentations of the methods; DSC: dice similarity coefficient; ICC: intra-class correlation coefficient. *Negative ICC (not used for calculating the overall mean ICC).

Because some methods (Cascade, Lesion-TOADS, LST-LGA, and LST-LPA) do not necessarily have to be trained, analyses were repeated on all subjects (n = 60) without training of the methods. This did not change the ranking of methods (data not shown). The average run time was shortest for Cascade (2 minutes), followed by kNN-TTP (10 minutes), LST-LPA (12 minutes), LST-LGA (25 minutes) and Lesion-TOADS (30 minutes).

### Variations in performance across scanners

For each method, we determined if the DSC (i.e. spatial correspondence with the reference standard) for each scanner differed relative to the other five scanners (Table 3). In this analysis, consistency of a method across scanners is reflected in small effect sizes. kNN-TTP showed the smallest variation in performance with the smallest effect sizes (range unstandardized beta coefficient: −0.06 to 0.01), followed by LST-LGA (−0.04 to 0.07), Cascade (−0.08 to 0.09), LST-LPA (−0.10 to 0.11) and Lesion-TOADS (−0.12 to 0.12). None of the effect sizes were significant after family wise error rate correction for multiple testing. Along the same lines, consistency of volumetric correspondence across scanners was assessed, by determining for each method the interaction between scanner and the relation between the assessed volume and the reference volume. Here we found a significant interaction for Lesion-TOADS on the Philips Ingenuity 3T scanner (family wise error rate corrected p < 0.05), indicating that performance was biased by scanner type. All other interactions were not significant (data not shown).

**Table 3:** Variation in performance across scanners by means of multiple linear regression analyses (n = 42; n = 7 per scanner).

Method	GE Signa HDxt 1.5T	GE Signa HDxt 3T	GE Discovery MR750 3T	Philips Ingenuity 3T	Philips Ingenia 3T	Philips Achieva 3T
Cascade	0.09 [−0.09; 0.27]	−0.06 [−0.24; 0.12]	−0.08 [−0.26; 0.10]	0.03 [−0.15; 0.21]	0.003 [−0.18; 0.18]	0.01[−0.17; 0.19]
kNN-TTP	0.01 [−0.08; 0.10]	−0.06 [−0.15; 0.03]	−0.03 [−0.12; 0.07]	0.02 [−0.08; 0.11]	0.03 [−0.06; 0.12]	0.03 [−0.06; 0.12]
Lesion-TOADS	0.12 [−0.08; 0.33]	0.04 [−0.17; 0.24]	−0.05 [−0.26; 0.16]	−0.12 [−0.33; 0.08]	0.10 [−0.11; 0.30]	−0.08 [−0.29; 0.12]
LST-LGA	0.02 [−0.11; 0.14]	−0.04 [−0.17; 0.09]	−0.03 [−0.16; 0.10]	−0.04 [−0.17; 0.09]	0.07 [−0.05; 0.20]	0.02 [−0.10; 0.15]
LST-LPA	0.06 [−0.07; 0.20]	−0.10 [−0.24; 0.03]	−0.09 [−0.23; 0.05]	−0.01 [−0.15; 0.13]	0.11 [−0.03; 0.24]	0.03 [−0.10; 0.17]

Data are represented as unstandardized beta coefficients with 95% confidence intervals. We assessed whether the DSC (as an outcome) depended on scanner (as a categorical variable with each scanner being compared to all other scanners as the reference) using linear regression analysis. A significant relation between a certain scanner and the DSC (family wise error rate corrected p-value of <0.05 using a Bonferroni correction) indicates that the performance (in terms of spatial correspondence with the reference segmentation) was biased for that segmentation method by the use of that scanner (compared to the other scanners). As can be seen in the table, no significant relations were seen for any of the methods.

### Performance of WMH segmentation methods for different WMH lesion loads

**Figure 2:**
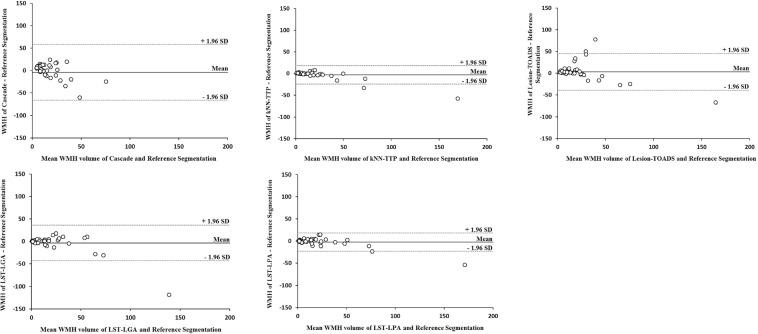
Bland Altman plots comparing WMH volume of each method versus the WMH volume of the reference segmentations. X-axis: mean WMH volume (in mL) of the automated and reference segmentations. Y-axis: difference (in mL) in WMH volume between the automated and reference segmentations. The lower (−1.96 SD) and upper (+1.96 SD) limits of agreement (dashed lines) and mean (straight line) are shown. A narrow width of the limits of agreement reflects a small amount of variation between the measurements of the reference and automated WMH segmentations. A positive difference on the y-axis is seen when WMH volume as measured by the automated method was larger than the reference WMH volume (i.e. overestimation). A negative difference on the y-axis is seen when WMH volume as measured by the automated method was smaller than the reference WMH volume (i.e. underestimation).

For all methods the DSC increased when Fazekas scores increased (see Table 4), as the DSC is particularly dependent on the absolute lesion load and the size of the individual lesions^18^. kNN-TTP and LST-LPA showed a good volumetric correspondence compared to the reference segmentations across all WMH lesion loads (see Table 4 and Supplementary Fig. 1). Also, variation in WMH volume measurements of these methods was small (i.e. narrow limits of agreement in the Bland Altman plots; see Fig. 2). Cascade, Lesion-TOADS and LST-LGA showed greater variation for different WMH lesion loads (i.e. wider limits of agreement in the Bland Altman plots, see Fig. 2). LST-LGA underestimated WMH volume at higher WMH lesion loads (see Fig. 2 and Supplementary Fig. 1). Cascade and Lesion-TOADS overestimated WMH volumes at lower WMH lesion loads, while Cascade underestimated WMH volumes at higher WMH lesion loads (see Fig. 2 and Supplementary Fig. 1).

**Table 4:** Performance of WMH segmentation methods for different WMH lesion loads.

Method	Fazekas scale	WMH volume reference	WMH volume method	ΔWMH	|ΔWMH|	DSC	ICC
Cascade	1	4 ± 4	12 ± 6	8 ± 6	8 ± 6	0.24 ± 0.16	0.02 (−0.12; 0.27)
	2	16 ± 10	18 ± 11	2 ± 12	10 ± 6	0.50 ± 0.15	0.31 (−0.16; 0.67)
	3	73 ± 61	26 ± 18	−47 ± 62	49 ± 60	0.54 ± 0.22	0.13 (−0.23; 0.67)
kNN-TTP	1	4 ± 4	5 ± 4	0.4 ± 1	0.9 ± 0.6	0.64 ± 0.10	0.91 (0.67; 0.97)
	2	16 ± 10	15 ± 9	−1 ± 3	3 ± 2	0.78 ± 0.06	0.96 (0.90; 0.99)
	3	73 ± 61	56 ± 41	−17 ± 22	18 ± 21	0.82 ± 0.06	0.92 (0.62; 0.99)
Lesion TOADS	1	4 ± 4	18 ± 20	13 ± 21	13 ± 21	0.35 ± 0.21	0.11 (−0.13; 0.43)
	2	16 ± 10	19 ± 11	3 ± 13	6 ± 12	0.61 ± 0.20	0.50 (0.08; 0.78)
	3	73 ± 61	53 ± 37	−20 ± 24	22 ± 22	0.77 ± 0.06	0.90 (0.49; 0.98)
LST-LGA	1	4 ± 4	4 ± 5	−0.3 ± 2	2 ± 2	0.47 ± 0.12	0.76 (0.46; 0.91)
	2	16 ± 10	15 ± 10	−0.4 ± 7	5 ± 5	0.61 ± 0.14	0.84 (0.63; 0.94)
	3	73 ± 61	53 ± 17	−20 ± 48	31 ± 40	0.70 ± 0.08	0.68 (−0.11; 0.94)
LST-LPA	1	4 ± 4	5 ± 5	0.3 ± 3	2 ± 2	0.49 ± 0.13	0.76 (0.45; 0.91)
	2	16 ± 10	14 ± 10	−2 ± 6	4 ± 4	0.64 ± 0.14	0.85 (0.60; 0.94)
	3	73 ± 61	62 ± 39	−11 ± 23	16 ± 18	0.78 ± 0.07	0.90 (0.53; 0.98)

Note: WMH, ΔWMH, |ΔWMH| and DSC are shown as means ± SD. ICC is shown as means (95% confidence interval).

ΔWMH: mean difference in WMH volume (mL) between the reference segmentations and segmentations of the methods.

|ΔWMH|: mean absolute difference in WMH volume (mL) between the reference segmentations and segmentations of the methods.

DSC: dice similarity coefficient; ICC: intra-class correlation coefficient.

Seventeen subjects had a Fazekas scale of 1, eighteen subjects had a Fazekas scale of 2 and seven subjects had a Fazekas scale of 3.

## Discussion

The current study is the first to investigate the performance of five freely available and fully automated segmentation methods in a multicenter dataset of patients with WMHs of presumed vascular origin. Overall, performance of methods in terms of spatial and volumetric correspondence varied markedly both within and across scanners, with kNN-TTP and LST-LPA being the most consistent and best performing methods. Our findings can serve as a guide for choosing a method. In Table 5, we have provided a qualitative recommendation for each method regarding several aspects when automatically segmenting WMHs based on the results described earlier.

**Table 5:** Considerations when choosing a method.

Method	Spatial correspondence	Volumetric correspondence	Lesion load	Different field strength	Different scanners	Computational Time
Cascade	−	−	−	−	+/−	++
kNN-TTP	+	++	+	+	+	+
Lesion TOADS	−	+/−	−	+	−	+/−
LST-LGA	−	+/−	−	+	+	+/−
LST-LPA	+/−	++	+	+/−	+/−	+

Note: ++: highly recommended; +: recommended; +/−; neutral; −: not recommended. Spatial correspondence: based on Dice’s Similarity Coefficient (DSC). Volumetric correspondence: based on intraclass correlation coefficient (ICC) and mean and mean absolute WMH volume differences. Lesion load: based on both spatial and volumetric correspondence with varying lesion loads. Different field strength: based on both spatial and volumetric correspondence on 1.5 Tesla compared to 3 Tesla MRI scanner of the same MRI vendor. Different scanners: based on the variation in performance across scanners, both in terms of spatial and volumetric correspondence. The (qualitative) recommendations were based on the results of the present study.

Many different automated methods currently exist to segment WMHs. Evaluation of these methods has mainly been performed in a single-center, single scanner setting, with variable performance across methods^6–8,10,11,17,19–41^. Some of these methods have also been assessed for scan-rescan reproducibility^6,8,18^, which is of particular importance when performing longitudinal research. However, since pooling of data across multiple centers is an important trend in small vessel disease research^42^, there also is a need for automated WMH segmentation methods that perform well across different scanners. Clearly, a multicenter setting with different scan vendors poses challenges, as the method cannot be tuned to one single scan protocol. The question is thus which methods perform robustly enough in such a setting, but this has been explored by few studies. A recent study, coordinated by our group, compared the performance of twenty methods, but in contrast to the present study, many of the tested methods are not freely available yet^43^. Two previous studies compared different linear and nonlinear classification techniques to segment WMHs of presumed vascular origin^44,45^. The important difference between these and the current is that they primarily focused on the optimal choice of classifiers for WMH segmentation, using a general preprocessing pipeline. By contrast, we evaluated some of the same classifiers as an integral part of a fully automated WMH segmentation method, where the classifier only partially determines the performance of the entire method.

We observed that for segmentation of WMHs of presumed vascular origin, performance of the five tested methods varied markedly, both within and across scanners. kNN-TTP and LST-LPA were the most consistent methods across scanners. kNN-TTP was also the best performing method within scanners with a DSC comparable to a manual segmentation as performed by a trained rater and an excellent ICC, whereas LST-LPA performed less with regard to spatial correspondence with the reference segmentations. This could be relevant when choosing a method to segment WMHs for further analysis where spatial information of WMHs is of particular importance (e.g. lesion symptom mapping^46^). By contrast, when analyzing WMH volumes as a primary outcome, both methods could be suitable.

All methods tended to slightly underestimate WMH volumes at higher lesion loads, but this was most prominent for LST-LGA and Lesion-TOADS. Lesion-TOADS and Cascade showed the lowest spatial and volumetric correspondence compared to the reference segmentation and especially performance of Lesion-TOADS also varied across scanners. A possible explanation for the differences in performance between methods, both within and across scanners, could be that some methods are more robust to sources of variation in MRI acquisition than others. In our study it is impossible to determine which MRI related factors contribute most to this variation. Future studies are therefore encouraged to determine these sources of variation and the relation to various methods. Another explanation within our study might be the variation in WMH volumes between scanners, which might have introduced variation caused by selection bias. Above all, our study highlights the need to further improve WMH segmentation methods. An important initiative was recently taken in the form of a WMH segmentation challenge^43^. In this challenge, new WMH segmentation methods were developed and evaluated on a multicenter dataset. The best performing method showed a similar DSC compared to kNN-TTP in the present study.

The number of subjects in our training set is relatively low: only eighteen subjects were used. The ability to train or optimize the included methods with only a limited number of training subjects can be considered a strength of the included approaches. It is often infeasible to acquire large amounts of training data (e.g. 100+ subjects). Our training set was composed in such a way that it included data from the six different scanners—located in two institutes—that were used in this study. This ensured a large amount of possible variation in the MRI data to be used for training (kNN-TTP) or post-hoc optimization (Cascade, Lesion-TOADS, LST-LGA, and LST-LPA) of the methods. Future studies could look into the optimal size and composition of the training set, possibly even further reducing the number of required training subjects. This would increase the applicability of these methods in other centers.

White matter lesions can also have a non-vascular etiology, like in multiple sclerosis (MS). White matter lesions in MS show a different load, morphology and distribution compared to WMHs of presumed vascular origin^5^. Nevertheless, evaluation of methods for segmentation of MS lesions can still be informative for WMH of vascular origin. In the field of MS, a previous study assessed the performance across scanners of Cascade, kNN-TTP, Lesion-TOADS, LST-LGA and LST-LPA^47^. This study showed the highest performance across scanners for kNN-TTP (DSC mean ± SD: 0.44 ± 0.14), followed by LST-LPA (0.37 ± 0.23), Lesion-TOADS (0.35 ± 0.18), LST-LGA (0.31 ± 0.23) and Cascade (0.26 ± 0.17). Although the etiology of MS lesions is different, the overall ranking of methods is comparable to the ranking in our study, with Cascade being the method with the worst performance. The overall performance for MS lesion segmentation of each method is however lower than in our study. This discrepancy can possibly be explained by the difference in white matter lesion load between the previous study in MS (WMH volume mean ± SD: 5 ± 7 mL) and our study (20 ± 9 mL). Particularly for the segmentation of multiple small lesions, the DSC can become relatively low.

The main strength of our study is that it allows a direct comparison in performance of these methods for multicenter use. To achieve this goal, we have constructed a high quality MRI dataset consisting of reference segmentations. A possible limitation could be the downsampling of the 3D FLAIR images, since performance of automated methods tends to be better at higher resolution. However, downsampling was necessary for a fair comparison across all scanners. Furthermore, manual segmentation of 3D FLAIR scans is more time consuming than 2D FLAIR scans. Another limitation could be the comparison of binary reference segmentations with binary automated segmentations (i.e. thresholding the initial probabilistic output of the automated methods). However, the alternative approach of creating probabilistic manual segmentations (e.g. by combining binary manual segmentations of the same subject performed by multiple raters into a single probabilistic segmentation) is very labor intensive. Moreover, it has limited added value over manual segmentation of a larger number of subjects. We have therefore invested in manual segmentations of more subjects in combination with determining optimal thresholds of the automated segmentations by using the training set. Another possible limitation of our study could be that we did not scan the same subject(s) on all six scanners. However, the aim of our study was not to assess (and quantify) the source of variation that could be introduced by using different MRI-scanners, but to determine the performance across scanners of widely used automated WMH segmentation methods in a dataset with different MRI-scanners that reflects general practice. A final limitation could be the selection of subjects for the present study. We chose to exclude subjects with severe motion artifacts and/or presence of large (sub)cortical brain infarcts. However, these brain abnormalities can often be observed in patients with WMH of presumed vascular origin and this could potentially lead to a different ranking in performance of the methods, as some methods might be more robust for these brain abnormalities. With regard to the design of the study and selection of methods, it could be argued that kNN-TTP is a supervised approach that uses fully annotated example data for training, whereas the other methods were only post hoc fine-tuned, which could have “favored” kNN-TTP as compared to the other methods. Yet, the counterargument would be that the training and test sets were kept fully separated in our study. Hence, the observation that a trained method, like kNN-TTP, outperformed the other methods would only strengthen the case for supervised methods in this application. In practice, such training takes only limited effort, as in our case the kNN-TTP was only offered a relatively low amount of training data (eighteen subjects).

In conclusion, performance of different methods for WMH segmentation varied markedly both within and across scanners. Our findings can serve as a guide for choosing a method and also highlight the importance to further improve and evaluate consistency of methods in a multicenter setting. Studies planning to segment WMHs from multicenter datasets should assess performance of their method of choice using a pilot sample of their data with manual segmentations.

## Materials and Methods

### Study population

Subjects with WMHs of presumed vascular origin (as defined by the STRIVE criteria)^48^ were selected from the TRACE-VCI study. This is a multicenter study on subjects with vascular cognitive impairment (VCI; n = 860) in the Netherlands and was described earlier^49^. In short, all patients that presented with cognitive complaints and vascular brain injury on MRI (i.e. possible VCI) were eligible to participate. Subjects scanned on six different MRI scanners were included. Four scanners were located at the Amsterdam University Medical Center (Amsterdam UMC), Amsterdam, the Netherlands (General Electric (GE) Signa HDxt 1.5T; GE Signa HDxt 3T; GE Discovery MR750 3T [General Electric Healthcare, Milwaukee, Wisconsin, USA] and Philips Ingenuity 3T [Philips Medical Systems, Best, the Netherlands]). Two scanners were located at the University Medical Center Utrecht (UMCU), Utrecht, the Netherlands (Philips Achieva 3T and Philips Ingenia 3T [Philips Medical Systems, Best, the Netherlands]). For the present study, ten subjects with varying WMH lesion load (Fazekas scale 1 to 3)^50^ were randomly selected per MRI scanner to represent the variation in WMH lesion load across the entire cohort. This led to inclusion of a total of 60 subjects (38 females, 22 males; age 68 ± 8 years). Compared to the entire cohort, there was no significant difference in age in the current study population (Student’s t-test; p > 0.05). There was a significant difference in gender (chi-square test; p < 0.05) with a relatively higher percentage of females in the current study population compared to the entire cohort^49^. Subjects with severe motion artifacts and/or presence of large (sub)cortical brain infarcts (less than 10% of the total cohort) were not considered for the present study. From the 60 subjects, we selected a training set of 18 subjects (i.e. three subjects per scanner; one randomly selected subject per Fazekas scale for each scanner) and a test set of 42 subjects (i.e. seven subjects per scanner). The training set and test set showed no significant difference in age (Student’s t test; p > 0.05), gender (chi-square test; p > 0.05) or WMH volume (Mann-Whitney U test; p > 0.05). The study was approved by the institutional review boards of the Amsterdam UMC and the UMCU (approval number 14-083/C). All procedures were in accordance with the ethical standards of the responsible committee on human experimentation (institutional and national) and with the Helsinki Declaration of 1975, as revised in 2013. All participating subjects provided written informed consent.

### MR imaging

All subjects were scanned using an MRI protocol that included a 3D T1-weighted and fluid-attenuated inversion recovery (FLAIR) sequence^49^. The MRI sequence parameters are shown in Table 6. To make a fair comparison across all MRI scanners, all 3D FLAIR scans from subjects who were scanned at the Amsterdam UMC, were resampled in the axial plane to better match the 2D FLAIR acquisitions from the UMCU. This was done using a linear interpolation tool in MeVisLab (MeVis Medical Solutions AG, Bremen, Germany), resulting in 3 mm slices with an in-plane resolution of 0.95–1.21 mm^51^.

**Table 6:** Overview of MRI sequence parameters for each scanner.

Center	Scanner vendor, type	Tesla	Sequence	Slices	TR (ms)	TE (ms)	TI (ms)	Voxel size (mm)
A	GE, Signa HDxt	1.5	3D T1	172	12.3	5.2	—	0.98 × 0.98 × 1.50
			3D FLAIR	128	6500	117	1987	1.21 × 1.21 × 1.30
A	GE, Signa HDxt	3	3D T1	176	7.8	3.0	—	0.94 × 0.94 × 1.00
			3D FLAIR	132	8000	126	2340	0.98 × 0.98 × 1.20
A	GE, Discovery MR750	3	3D T1	176	8.2	3.2	—	0.94 × 0.94 × 1.00
			3D FLAIR	160	8000	130	2340	0.98 × 0.98 × 1.20
A	Philips, Ingenuity	3	3D T1	180	9.9	4.6	—	0.87 × 0.87 × 1.00
			3D FLAIR	321	4800	279	1650	1.04 × 1.04 × 0.56
B	Philips, Achieva	3	3D T1	192	7.9	4.5	—	1.00 × 1.00 × 1.00
			2D FLAIR	48	11000	125	2800	0.96 × 0.95 × 3.00
B	Philips, Ingenia	3	3D T1	192	7.9	4.5	—	1.00 × 1.00 × 1.00
			2D FLAIR	48	11000	125	2800	0.96 × 0.95 × 3.00

Note: A = Amsterdam University Medical Center; B = Utrecht University Medical Center; TR = repetition time; TE = echo time; TI = inversion time.

### Reference segmentations

WMH reference segmentations were constructed as reference data for training and testing the automated WMH segmentation methods. The reference segmentations were obtained for all subjects, prior to and without knowledge of the results of the automated segmentation methods, using the following procedure. An in-house developed MeVisLab (MeVis Medical Solutions AG, Bremen, Germany) tool was used to semi-automatically delineate the contour of WMHs on all axial slices^46,51^. In short, WMHs were segmented using an iso-contouring technique. Contours were converted into binary segmentation masks by including all voxels having a (sub)voxel volume of at least 20% within the contour. This threshold value was chosen by visual comparison of images thresholded with values between 0 and 100% (intervals of 5%). All reference segmentations were constructed by a single rater (RH). To assess inter-rater reliability of the reference segmentations, JMB constructed reference segmentations on a subset of twenty subjects by using the same semi-automatic procedure. To assess intra-rater reliability of the reference segmentations, RH constructed a second segmentation on a subset of twenty subjects.

### Automated WMH segmentation methods

For the present study, we included methods that were fully-automated and freely available for academic research: Cascade, kNN-TTP, Lesion-TOADS, LST-LGA, and LST-LPA. All methods were ran on FLAIR and 3D T1-weighted MR-images of all subjects to obtain WMH segmentations. Default settings were used as much as possible. The training set of subjects (n = 18) was used to train and tune each of the methods (i.e. to determine optimal thresholds). For Cascade, we ran the segmentation algorithm on the training set while changing the two main parameters (lower threshold and upper threshold: {0.05, 0.075, 0.100, …, 1.00})^15,16^. We then chose the parameter combination that generated the highest DSC in the training set (in the current study: lower threshold = 0.95; upper threshold = 0.975). A similar approach was used to derive the optimal parameter settings for LST-LGA (parameters kappa {0.05, 0.10, …, 1.00} and lesion probability threshold {0.05, 0.10, …, 1.00}; optimal settings for kappa: 0.25 and lesion probability threshold of 0.2)^10^. For LST-LPA and kNN-TTP only the lesion probability threshold was tuned {0.05, 0.10, …, 1.00}, resulting in optimal values of 0.3 for LST-LPA and 0.35 for kNN-TTP^17^. Because in kNN-TTP, the reference data are actively used in every run of the algorithm, a leave-one-out cross-validation was used to optimize kNN-TTP parameters to ensure independence of the evaluation^17^. We did not exclude specific brain regions (such as the brain stem or basal ganglia where often higher false positive rates can be observed) from the analyses, since the aim of our study was to evaluate methods using their own processing. For a detailed overview of the workflow used for each method, see the Supplementary Information.

### Statistical analysis

All automated WMH segmentation methods were evaluated on the test set (n = 42; i.e. 7 subjects per scanner). Several evaluation metrics currently exist to evaluate performance of WMH segmentation methods, each with their own advantages and disadvantages (for an overview see^52^). For the present study, we chose frequently used evaluation metrics that have been used in recent comparative studies on WMH segmentation^8,47^.

#### Quality assessment

We evaluated all methods qualitatively by visually comparing the output of each method with the reference segmentations. Next, we evaluated all methods quantitatively by calculating false positive (FP) volumes (in mL) and false negative (FN) volumes (in mL) of the WMH segmentations of each method using the reference segmentations.

#### Performance within scanners

The performance of each method was assessed per scanner by measuring: (a) the spatial (i.e. voxel-wise) correspondence with the reference segmentations by using the DSC; (b) the volumetric correspondence with the reference WMH volumes by using the ICC (two-way mixed model with absolute agreement after log-transforming WMH volumes because of non-normal distribution); (c) the mean differences and mean absolute differences between WMH volumes of each method and the reference WMH volumes. Because specific methods (Cascade, Lesion-TOADS, LST-LGA, and LST-LPA) do not necessarily have to be trained, performance was also determined in secondary analyses on all subjects (n = 60) without training of the methods.

#### Mean performance across scanners

The mean performance of each method across scanners was determined by averaging the mean DSC, ICC and absolute volume differences of each scanner.

#### Variations in performance across scanners

To investigate the variation in performance across scanners of each method, we performed the following two analyses:

- For each method, we assessed whether the DSC (as an outcome) depended on scanner (as a categorical variable with each scanner being compared to all other scanners as the reference) using linear regression analysis. This resulted in a unstandardized beta coefficient with 95% confidence intervals for each scanner. A significant relation between a certain scanner and the DSC (family wise error rate corrected p-value of <0.05 using a Bonferroni correction) indicates that the performance (in terms of spatial correspondence with the reference segmentation) was biased by the use of that scanner (compared to the other scanners).
- For each method, we assessed whether the relation between the reference WMH volumes (as an outcome) and WMH volumes of the automated WMH segmentation method (as a determinant) depended on scanner (as a categorical variable with each scanner being compared to all other scanners as the reference) by using linear regression analyses. Because of non-normal distribution, WMH volumes of each method and the reference WMH volumes were log-transformed. A significant interaction between the log transformed WMH volume of a method and a certain scanner (family wise error rate corrected p-value of <0.05), indicates that performance of that method (in terms of volumetric correspondence with the reference segmentation) was biased by the use of that scanner (compared to the other scanners).

#### Performance for different WMH lesion loads

In addition, the MRI scans of all subjects were stratified based on the Fazekas scale (Fazekas scale 1/2/3: n = 17/n = 18/n = 7). We then assessed whether the performance of each method was dependent on the WMH lesion load (i.e. Fazekas scale) using DSC, ICC and mean (absolute) volume differences. In addition, Bland-Altman plots were made to compare WMH volume of each method with the reference WMH volumes^53^. Bland Altman plots provide a graphical representation of the amount of variation from the mean when comparing WMH volumes of the WMH segmentation methods and the reference segmentations. In these plots, a narrow width of the limits of agreement reflects a small amount of variation between WMH volumes of the WMH segmentation methods and the reference segmentations. The difference between WMH volumes of the WMH segmentation methods and the reference segmentation reflects over- or underestimation of the WMH segmentation methods. Both a change in the direction of WMH volume differences (i.e. positive or negative differences) as well as the distribution of WMH volume differences (narrow or wide) for different WMH lesion loads, can reflect performance of a WMH segmentation method to be dependent on the WMH lesion load.

## Supplementary information

Supplementary Information

## Data Availability

The data that support the findings of this study are available from the final author, upon reasonable request.

## References

[1] Carrillo MC, Bain LJ, Frisoni GB, Weiner MW (2012). Worldwide Alzheimer’s disease neuroimaging initiative. Alzheimers. Dement..

[2] Williamson, J. D. *et al*. The Action to Control Cardiovascular Risk in Diabetes Memory in Diabetes Study (ACCORD-MIND): Rationale, Design, and Methods. *Am*. *J*. *Cardiol*. **99** (2007). 10.1016/j.amjcard.2007.03.029 17599421

[3] Mueller SG (2005). Ways toward an early diagnosis in Alzheimer’s disease: The Alzheimer’s Disease Neuroimaging Initiative (ADNI). Alzheimer’s Dement..

[4] De Guio F (2016). Reproducibility and variability of quantitative magnetic resonance imaging markers in cerebral small vessel disease. J. Cereb. Blood Flow Metab..

[5] Caligiuri ME (2015). Automatic Detection of White Matter Hyperintensities in Healthy Aging and Pathology Using Magnetic Resonance Imaging: A Review. Neuroinformatics.

[6] Jain S (2015). Automatic segmentation and volumetry of multiple sclerosis brain lesions from MR images. NeuroImage Clin..

[7] Ghafoorian M (2016). Automated detection of white matter hyperintensities of all sizes in cerebral small vessel disease. Med. Phys..

[8] Griffanti L (2016). BIANCA (Brain Intensity AbNormality Classification Algorithm): A new tool for automated segmentation of white matter hyperintensities. Neuroimage.

[9] Bowles C (2016). Pseudo-healthy image synthesis for white matter lesion segmentation. Lecture Notes in Computer Science (including subseries Lecture Notes in Artificial Intelligence and Lecture Notes in Bioinformatics).

[10] Schmidt P (2012). An automated tool for detection of FLAIR-hyperintense white-matter lesions in Multiple Sclerosis. Neuroimage.

[11] Shiee N (2010). A topology-preserving approach to the segmentation of brain images with multiple sclerosis lesions. Neuroimage.

[12] Qin C (2018). A large margin algorithm for automated segmentation of white matter hyperintensity. Pattern Recognit..

[13] Guerrero R (2018). White matter hyperintensity and stroke lesion segmentation and differentiation using convolutional neural networks. NeuroImage Clin..

[14] Ling Yifeng, Jouvent Eric, Cousyn Louis, Chabriat Hugues, De Guio François (2018). Validation and Optimization of BIANCA for the Segmentation of Extensive White Matter Hyperintensities. Neuroinformatics.

[15] Damangir S (2012). Multispectral MRI segmentation of age related white matter changes using a cascade of support vector machines. J. Neurol. Sci..

[16] Damangir S (2017). Reproducible segmentation of white matter hyperintensities using a new statistical definition. Magn. Reson. Mater. Physics, Biol. Med..

[17] Steenwijk MD (2013). Accurate white matter lesion segmentation by k nearest neighbor classification with tissue type priors (kNN-TTPs). NeuroImage. Clin..

[18] Admiraal-Behloul F (2005). Fully automatic segmentation of white matter hyperintensities in MR images of the elderly. Neuroimage.

[19] Admiraal-Behloul F (2005). Fully automatic segmentation of white matter hyperintensities in {MR} images of the elderly. Neuroimage.

[20] Anbeek P, Vincken KL, Van Osch MJP, Bisschops RHC, Van Der Grond J (2004). Probabilistic segmentation of white matter lesions in MR imaging. Neuroimage.

[21] Beare R (2009). Development and validation of morphological segmentation of age-related cerebral white matter hyperintensities. Neuroimage.

[22] Brickman AM (2011). Quantitative approaches for assessment of white matter hyperintensities in elderly populations. Psychiatry Res. - Neuroimaging.

[23] de Boer R (2009). White matter lesion extension to automatic brain tissue segmentation on MRI. Neuroimage.

[24] Erus G, Zacharaki EI, Davatzikos C (2014). Individualized statistical learning from medical image databases: Application to identification of brain lesions. Med. Image Anal..

[25] Gibson E, Gao F, Black SE, Lobaugh NJ (2010). Automatic segmentation of white matter hyperintensities in the elderly using FLAIR images at 3T. J. Magn. Reson. Imaging.

[26] Herskovits EH, Bryan RN, Yang F (2008). Automated Bayesian segmentation of microvascular white-matter lesions in the ACCORD-MIND study. Adv. Med. Sci..

[27] Iorio, M. *et al*. White matter hyperintensities segmentation: A new semi-automated method. *Front*. *Aging Neurosci*. **5** (2013). 10.3389/fnagi.2013.00076 PMC3857525 24339815

[28] Ithapu V (2014). Extracting and summarizing white matter hyperintensities using supervised segmentation methods in Alzheimer’s disease risk and aging studies. Hum. Brain Mapp..

[29] Khayati R, Vafadust M, Towhidkhah F, Nabavi M (2008). Fully automatic segmentation of multiple sclerosis lesions in brain MR FLAIR images using adaptive mixtures method and markov random field model. Comput. Biol. Med..

[30] Lao Z (2008). Computer-Assisted Segmentation of White Matter Lesions in 3D MR Images Using Support Vector Machine. Acad. Radiol..

[31] Moeskops P (2017). Evaluation of a deep learning approach for the segmentation of brain tissues and white matter hyperintensities of presumed vascular origin in MRI. NeuroImage Clin..

[32] Ramirez J (2011). Lesion Explorer: A comprehensive segmentation and parcellation package to obtain regional volumetrics for subcortical hyperintensities and intracranial tissue. Neuroimage.

[33] Rincón M (2017). Improved Automatic Segmentation of White Matter Hyperintensities in MRI Based on Multilevel Lesion Features. Neuroinformatics.

[34] Sajja BR (2006). Unified approach for multiple sclerosis lesion segmentation on brain MRI. Ann. Biomed. Eng..

[35] Simões R (2013). Automatic segmentation of cerebral white matter hyperintensities using only 3D FLAIR images. Magn. Reson. Imaging.

[36] Smart SD, Firbank MJ, O’Brien JT (2011). Validation of automated white matter hyperintensity segmentation. J. Aging Res..

[37] Tsai JZ (2014). Automated segmentation and quantification of white matter hyperintensities in acute ischemic stroke patients with cerebral infarction. PLoS One.

[38] Wang R (2015). Automatic segmentation and volumetric quantification of white matter hyperintensities on fluid-attenuated inversion recovery images using the extreme value distribution. Neuroradiology.

[39] Wang R (2014). Automatic segmentation and quantitative analysis of white matter hyperintensities on FLAIR images using trimmed-likelihood estimator. Acad. Radiol..

[40] Wu Y (2006). Automated segmentation of multiple sclerosis lesion subtypes with multichannel MRI. Neuroimage.

[41] Zhong, Y., Utriainen, D., Wang, Y., Kang, Y. & Haacke, E. M. Automated White Matter Hyperintensity Detection in Multiple Sclerosis Using 3D T2 FLAIR. *Int*. *J*. *Biomed*. *Imaging* **2014** (2014). 10.1155/2014/239123 PMC4130152 25136355

[42] Dichgans M (2016). METACOHORTS for the study of vascular disease and its contribution to cognitive decline and neurodegeneration: An initiative of the Joint Programme for Neurodegenerative Disease Research. Alzheimer’s and Dementia.

[43] Kuijf Hugo J., Casamitjana Adria, Collins D. Louis, Dadar Mahsa, Georgiou Achilleas, Ghafoorian Mohsen, Jin Dakai, Khademi April, Knight Jesse, Li Hongwei, Llado Xavier, Biesbroek J. Matthijs, Luna Miguel, Mahmood Qaiser, McKinley Richard, Mehrtash Alireza, Ourselin Sebastien, Park Bo-Yong, Park Hyunjin, Park Sang Hyun, Pezold Simon, Puybareau Elodie, De Bresser Jeroen, Rittner Leticia, Sudre Carole H., Valverde Sergi, Vilaplana Veronica, Wiest Roland, Xu Yongchao, Xu Ziyue, Zeng Guodong, Zhang Jianguo, Zheng Guoyan, Heinen Rutger, Chen Christopher, van der Flier Wiesje, Barkhof Frederik, Viergever Max A., Biessels Geert Jan, Andermatt Simon, Bento Mariana, Berseth Matt, Belyaev Mikhail, Cardoso M. Jorge (2019). Standardized Assessment of Automatic Segmentation of White Matter Hyperintensities and Results of the WMH Segmentation Challenge. IEEE Transactions on Medical Imaging.

[44] Dadar M (2017). Performance comparison of 10 different classification techniques in segmenting white matter hyperintensities in aging. Neuroimage.

[45] Samaille Thomas, Fillon Ludovic, Cuingnet Rémi, Jouvent Eric, Chabriat Hugues, Dormont Didier, Colliot Olivier, Chupin Marie (2012). Contrast-Based Fully Automatic Segmentation of White Matter Hyperintensities: Method and Validation. PLoS ONE.

[46] Biesbroek JM (2016). Impact of Strategically Located White Matter Hyperintensities on Cognition in Memory Clinic Patients with Small Vessel Disease. PLoS One.

[47] de Sitter A (2017). Performance of five research-domain automated WM lesion segmentation methods in a multi-center MS study. Neuroimage.

[48] Wardlaw JM (2013). Neuroimaging standards for research into small vessel disease and its contribution to ageing and neurodegeneration. The Lancet Neurology.

[49] Boomsma JMF (2017). Vascular Cognitive Impairment in a Memory Clinic Population: Rationale and Design of the ‘Utrecht-Amsterdam Clinical Features and Prognosis in Vascular Cognitive Impairment’ (TRACE-VCI) Study. JMIR Res. Protoc..

[50] Fazekas F, Chawluk JB, Alavi A (1987). MR signal abnormalities at 1.5 T in Alzheimer’s dementia and normal aging. American Journal of Neuroradiology.

[51] Ritter F (2011). Medical image analysis. IEEE Pulse.

[52] Taha, A. A. & Hanbury, A. Metrics for evaluating 3D medical image segmentation: Analysis, selection, and tool. *BMC Med*. *Imaging* **15** (2015). 10.1186/s12880-015-0068-x PMC4533825 26263899

[53] Martin Bland J, Altman D (1986). Statistical Methods for Assessing Agreement Between Two Methods of Clinical Measurement. Lancet.

