# Supplementary material for: Performance of five automated white matter hyperintensity segmentation methods in a multicenter dataset
Source: Sci Rep. 2019 Nov 14;9:16742. doi: 10.1038/s41598-019-52966-0 (PMC6856351; doi:10.1038/s41598-019-52966-0)
Supplement: Supplementary file 1 — Supplementary Information [file 41598_2019_52966_MOESM1_ESM.docx]

**Supplementary Information**

**Performance of five automated white matter hyperintensity segmentation methods in a multicenter dataset**

**Author names and affiliations:**

1. Rutger Heinen*, MD^1^
2. Martijn D. Steenwijk, PhD^2,3^
3. Frederik Barkhof, MD, PhD^3,4^
4. J. Matthijs Biesbroek, MD, PhD^1^
5. Wiesje M. van der Flier, PhD^5,6^
6. Hugo J. Kuijf, PhD^7^
7. Niels D. Prins, MD, PhD^5^
8. Hugo Vrenken, PhD^2,3^
9. Geert Jan Biessels, MD, PhD^1^
10. Jeroen de Bresser, MD, PhD^8^

On behalf of the TRACE-VCI study group.

* Corresponding author.

^1^ Department of Neurology and Neurosurgery, UMC Utrecht Brain Center, University Medical Center Utrecht, Utrecht University, Utrecht, the Netherlands.

^2^ Department of Anatomy and Neurosciences, Amsterdam Neuroscience, Vrije Universiteit Amsterdam, Amsterdam UMC, Amsterdam, the Netherlands.

^3^ Department of Radiology and Nuclear Medicine, Amsterdam Neuroscience, Vrije Universiteit Amsterdam, Amsterdam UMC, Amsterdam, the Netherlands.

**Materials and Methods**

**Cascade**

Cascade (version 1.1; available via <https://github.com/Damangir/Cascade> (Damangir et al., 2017, 2012) is an unsupervised method based on a proposed statistical definition of WMH. It uses a single node support vector machine (SVM) to preselect tissues that show differences in white matter (WM) intensity compared to normal gray and white matter. These changes in WM are then tested against a statistical definition of WMH. By applying a threshold (α), a selection of WMH is made. A probability map is then generated by applying a morphological filter and removing small lesions. Finally, a binary WMH segmentation is generated by applying another threshold (based on the lesion p-value). A recent paper on Cascade suggest any form of preprocessing can be done as long as all input images for Cascade have been registered and corrected for inhomogeneity and initial brain segmentation in WM, gray matter (GM) and cerebrospinal fluid (CSF) has been performed (Damangir et al., 2017). To minimize differences in preprocessing between other methods (i.e. Lesion-TOADS), we chose to use the following preprocessing. Bias correction of both the T1 and FLAIR images was performed using SPM12 (default settings). Brain segmentation was performed using CAT12 (version r864; <http://dbm.neuro.uni-jena.de/cat/>). CAT12 is a SPM tool that uses voxel-based morphometry to classify GM, WM and CSF. Internal Cascade tools were then used to refined the initial brain segmentation of CAT12 using the bias-corrected FLAIR image. During refining, intensities of the FLAIR image were used to correct for misclassified WMH (e.g. as GM instead of WMH). Next, the refined brain tissue segmentation and bias-corrected T1 were registered to the original, bias-corrected FLAIR image using Elastix (Klein et al., 2010). These three images were then used for WMH segmentation by Cascade. To determine the optimal settings, we chose the settings that generated the highest DSC in the training set (in the current study: lower threshold = 0.95; upper threshold = 0.975; rest of the settings were kept at default).

**kNN-TTP**

kNN-TTP is a supervised method based on a k-nearest neighbor algorithm, combined with tissue type priors derived from healthy controls to segment WMHs (Steenwijk et al., 2013). WMHs are segmented by comparing each voxel of the labelled training set with new data of the test set. The lesion-probability map is then converted to a binary WMH segmentation by applying a lesion probability threshold. For our study, we used the original T1 and FLAIR images as well as the brain masks (that were acquired as described earlier). kNN-TTP uses its own standard preprocessing pipeline (for details see (Steenwijk et al., 2013)). After preprocessing, kNN-TTP extracts eight features for kNN classification: FLAIR and T1 signal intensity, MNI-normalized spatial coordinates *x*, *y* and *z*, and tissue type probabilities pCSF, pGM and pWM. These features are normalized using variance scaling to account for different ranges for different features. Next, classification of each voxel was performed by determining the fraction of *k*-nearest neighbors that were labeled as being a lesion in the training set. For our study, *k* was set to 40 (Anbeek et al., 2008). The output of kNN-TTP is a probabilistic lesion map. Finally, a threshold of 0.45 was used to obtain a binary WMH segmentation. This threshold was chosen by determining the best DSC in the training set using thresholds between 0 and 1 (intervals of 0.05).

**Lesion-TOADS**

Lesion Topology-preserving Anatomical Segmentation (Lesion-TOADS; version 1.9) is an unsupervised method that is available as a plug-in for the MIPAV software (<http://mipav.cit.nih.gov/>) (Shiee et al., 2010). Lesion-TOADS uses an algorithm for fuzzy classification of image intensities, using a combination of topological and statistical atlases. An additional WMH lesion class is added to the brain segmentation model, using the same spatial prior as WM. Next, WMH and WM are separated by selecting, inside the grouped region, whichever has the higher membership value. The distances to certain areas (e.g. distance to ventricles) are used to correct for possible false positives. For Lesion-TOADS the following preprocessing was performed. First, FLAIR images were registered to T1 using Elastix. Next, bias correction was performed using SPM12 (default settings) on both T1 and coregistered FLAIR images. Subsequently, a brain mask was obtained using CAT12 (and manually corrected if needed). The resulting brain mask was used to skull strip both T1 and coregistered FLAIR images. The coregistered, bias corrected and skull-stripped T1 and FLAIR images were then used by Lesion-TOADS. For our current study, default settings were used. To compare the WMH segmentation of Lesion-TOADS (in 3D T1 space) with the manual segmentations (in FLAIR space), the following procedure was performed. First, Elastix was used to perform a rigid registration of the 3D T1 to the FLAIR image (Klein et al., 2010). Next, the resulting translation parameters were used to transform the WMH map to FLAIR space. A final threshold of 0.35 was applied to obtain a binary WMH map. This threshold was chosen by determining the best DSC in the training set using thresholds between 0 and 1 (intervals of 0.05).

**LST (LGA and LPA)**

LST-LGA is part of the Lesion Segmentation Tool (version 2.0.14), a toolbox that can be downloaded and subsequently implemented in SPM12 ([www.statisticalmoddeling.de/lst/html](http://www.statisticalmoddeling.de/lst/html)) (Schmidt et al., 2012). It segments the T1 image into three main tissue classes (GM, WM and CSF). These segmentations are then combined with the coregistered FLAIR intensities in order to calculate WMH (‘lesion belief’ maps). By thresholding these maps with a pre-chosen initial threshold (kappa), an initial binary WMH map is obtained, which is subsequently grown along voxels that are hyperintense on the FLAIR image. The result is a lesion probability map.

LST-LPA is part of the LST toolbox (version 2.0.14) and consists of a binary classifier in the form of a logistic regression model trained on the data of 53 subjects with multiple sclerosis with severe lesion patterns (<http://www.applied-statistics.de/lst.html>). Data were obtained at the Department of Neurology, Technische Universität München, Munich, Germany. As covariates for this model a similar WMH (‘lesion belief’) map as for the LST-LGA was used as well as a spatial covariate that takes into account voxel specific changes in WMH probability. Parameters of this model fit are used to segment WMH in new images by providing an estimate for the WMH probability for each voxel. This results in a WMH probability map.

No preprocessing was performed, as LST uses internal SPM preprocessing tools. To compare the probabilistic WMH segmentation of LST (in 3D T1 space) with the binary manual segmentations (in FLAIR space), Elastix was used to perform a rigid registration of the 3D T1 to the FLAIR image (Klein et al., 2010). Next, the WMH probability map was transformed to FLAIR space. A final threshold of 0.2 was applied for LST-LGA and 0.3 for LST-LPA to obtain a binary WMH map. These thresholds were chosen by determining the best DSC in the training set using thresholds between 0 and 1 (intervals of 0.05). For LST-LG A we tested different thresholds for kappa that were between 0 and 1 (intervals of 0.05) and determined the optimal threshold (0.25) with the highest observed DSC in the training set.

**References**

Anbeek, P., Vincken, K., Viergever, M., 2008. Automated MS-lesion segmentation by k-nearest neighbor classification. Midas J. 1–8.

Damangir, S., Manzouri, A., Oppedal, K., Carlsson, S., Firbank, M.J., Sonnesyn, H., Tysnes, O.B., O’Brien, J.T., Beyer, M.K., Westman, E., Aarsland, D., Wahlund, L.O., Spulber, G., 2012. Multispectral MRI segmentation of age related white matter changes using a cascade of support vector machines. J. Neurol. Sci. 322, 211–216. https://doi.org/10.1016/j.jns.2012.07.064

Damangir, S., Westman, E., Simmons, A., Vrenken, H., Wahlund, L.O., Spulber, G., 2017. Reproducible segmentation of white matter hyperintensities using a new statistical definition. Magn. Reson. Mater. Physics, Biol. Med. 30, 227–237. https://doi.org/10.1007/s10334-016-0599-3

Klein, S., Staring, M., Murphy, K., Viergever, M.A., Pluim, J.P.W., 2010. Elastix: A toolbox for intensity-based medical image registration. IEEE Trans. Med. Imaging 29, 196–205. https://doi.org/10.1109/TMI.2009.2035616

Schmidt, P., Gaser, C., Arsic, M., Buck, D., F??rschler, A., Berthele, A., Hoshi, M., Ilg, R., Schmid, V.J., Zimmer, C., Hemmer, B., M??hlau, M., 2012. An automated tool for detection of FLAIR-hyperintense white-matter lesions in Multiple Sclerosis. Neuroimage 59, 3774–3783. https://doi.org/10.1016/j.neuroimage.2011.11.032

Shiee, N., Bazin, P.-L., Ozturk, A., Reich, D.S., Calabresi, P.A., Pham, D.L., 2010. A topology-preserving approach to the segmentation of brain images with multiple sclerosis lesions. Neuroimage 49, 1524–1535. https://doi.org/10.1016/j.neuroimage.2009.09.005

Steenwijk, M.D., Pouwels, P.J.W., Daams, M., van Dalen, J.W., Caan, M.W.A., Richard, E., Barkhof, F., Vrenken, H., 2013. Accurate white matter lesion segmentation by k nearest neighbor classification with tissue type priors (kNN-TTPs). NeuroImage. Clin. 3, 462–9. https://doi.org/10.1016/j.nicl.2013.10.003

**Supplementary Fig 1**

**Supplementary Fig. 1.** WMH volume measurements of each method compared to reference WMH segmentation.


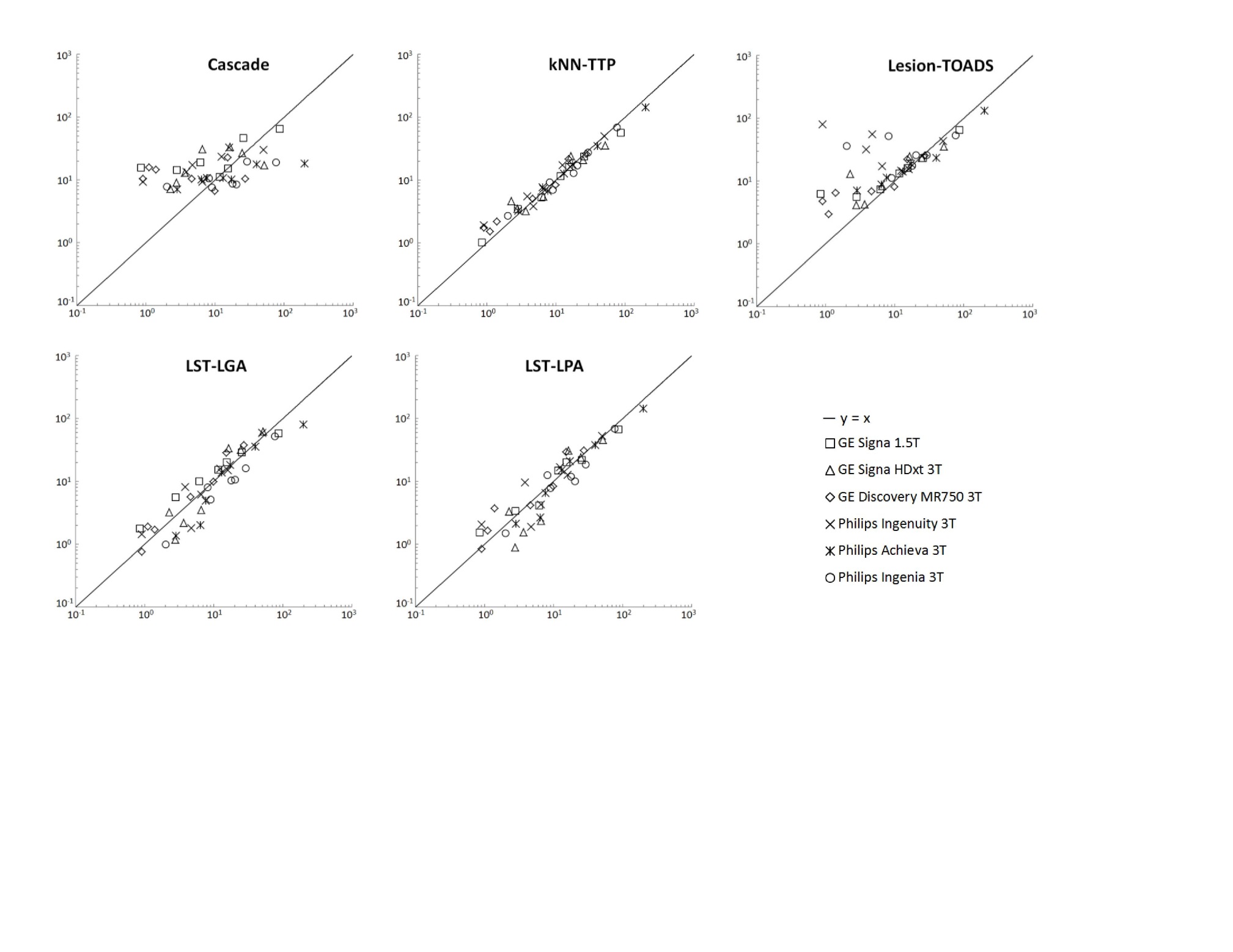


Note: WMH volume measurements (in mL) of WMH segmentation methods (left to right, up to down: Cascade, kNN-TTP, Lesion-TOADS, LST-LGA and LST-LPA) shown on x-axis against the mean WMH volume measurements (in mL) of the reference segmentations on y-axis. Note: log scale is used for both axes.
